# Renalase knockdown inhibits proliferation of mouse satellite cells

**DOI:** 10.1007/s11033-026-11803-0

**Published:** 2026-04-17

**Authors:** Yuri Kato, Katsuyuki Tokinoya, Kai Aoki, Kazuhiro Takekoshi

**Affiliations:** 1 Graduate School of Comprehensive Human Sciences, University of Tsukuba, 1-1-1 Tennodai, Tsukuba, Ibaraki 305-8574 Japan; 2 Research Fellowship for Young Scientists, Japan Society for the Promotion of Science, 5-3-1 Kojimachi, Chiyodaku, Tokyo 102-0083 Japan; 3 College of Education, Yokohama National University, 79-2 Tokiwadai, Hodogaya-Ku, Yokohama, Kanagawa 240-8501 Japan; 4 Department of Health and Nutrition, Faculty of Health Science, Niigata University of Health and Welfare, 1398 Shimami-Cho, Kita-Ku, Niigata, 950-3198 Japan; 5 Faculty of Medicine, Institute of Medicine, University of Tsukuba, 1-1-1 Tennodai, Tsukuba, Ibaraki 305-8574 Japan

**Keywords:** Renalase, Skeletal muscle, Satellite cell, Cell proliferation, Self-renewal, Akt

## Abstract

**Background:**

Skeletal muscle regeneration is mediated by skeletal muscle stem cells, known as satellite cells. Satellite cell proliferation is regulated by various secreted factors, including exercise-induced cytokines. Renalase is a protein that promotes cell proliferation through activation of intracellular signaling pathways in tumor cells. However, the role of renalase in satellite cell proliferation remains unclear. This study aimed to elucidate the role of renalase in satellite cell proliferation.

**Methods and Results:**

Satellite cells were isolated from the extensor digitorum longus (EDL) muscles of male mice. Renalase expression was suppressed using an adenoviral vector expressing short hairpin RNA (shRNA). Renalase knockdown significantly suppressed cell numbers, particularly reducing the Pax7⁺/MyoD⁺ cell population. In addition, the numbers of Ki67-positive and EdU-positive cells were significantly reduced, and the expression of genes involved in cell-cycle progression was significantly decreased. Furthermore, the phosphorylation levels of ERK1/2 and Akt, key signaling molecules involved in cell proliferation, were significantly reduced following renalase knockdown.

**Conclusion:**

These results indicate that renalase is essential for satellite cell proliferation and that renalase knockdown inhibits satellite cell proliferation by suppressing the Akt–mTOR and ERK1/2 signaling pathways.

**Supplementary Information:**

The online version contains supplementary material available at 10.1007/s11033-026-11803-0.

## Introduction

Skeletal muscle is a highly regenerative tissue that can repair itself promptly after injury. This regenerative capacity is mediated by skeletal muscle stem cells, known as satellite cells. Satellite cells remain in a quiescent state and express the transcription factor Pax7 under normal conditions. When skeletal muscle is injured, these cells become activated, upregulate the myoblast determination protein 1 (MyoD), and enter the cell cycle to proliferate. Subsequently, they downregulate Pax7 expression and express the myogenic regulatory factor Myogenin, thereby exiting the cell cycle, committing to differentiation, and fusing to form new myofibers [1–3]. In addition, a subset of proliferating satellite cells returns to a quiescent state, contributing to the maintenance of the satellite cell pool [4, 5]. Therefore, it is essential to elucidate the molecular mechanisms that regulate satellite cell proliferation to understand skeletal muscle regeneration. 

Satellite cell proliferation is regulated by various secreted factors [6, 7]. For example, fibroblast growth factor-2 (FGF-2) promotes satellite cell proliferation through activation of p38α/β mitogen-activated protein kinase (MAPK) signaling [8]. In addition, FGF-2 plays a critical role in cell-cycle progression from the G1 to S phase via activation of extracellular signal-regulated kinase 1/2 (ERK1/2) [9, 10]. Furthermore, insulin-like growth factor-1 (IGF-1) promotes satellite cell proliferation through activation of the phosphatidylinositol 3′-kinase (PI3K)/Akt signaling pathway [11]. These molecules are also recognized as exercise-induced cytokines [12]. Moreover, exercise has been reported to activate and increase satellite cell number [13–16]. Collectively, these findings indicate that signaling mediated by secreted factors plays a crucial role in satellite cell proliferation regulation.

Renalase is a secreted protein originally identified as a flavin adenine dinucleotide (FAD)-dependent amine oxidase [17]. However, subsequent studies have revealed that extracellular renalase functions not as an enzyme but as a cytokine [18–21]. Extracellular renalase activates intracellular signaling pathways, including p38 MAPK, ERK1/2, and Akt, via its receptor, plasma membrane Ca^2^⁺-ATPase 4b (PMCA4b), thereby exerting cytoprotective effects [20–23]. Furthermore, in tumor cells, increased renalase expression promotes cell proliferation through STAT3 activation [24–26].

Exercise increases circulating renalase levels and its expression in skeletal muscle [27–30]. In addition, exercise-induced upregulation of renalase expression in skeletal muscle has been associated with Akt activation [28]. Based on these findings, renalase is likely to contribute to the proliferation of satellite cells, the stem cells of skeletal muscle, through activation of intracellular signaling pathways. Indeed, published RNA-seq data (GSE121589) demonstrate the presence of renalase transcripts in satellite cells [31]. However, no studies to date have specifically investigated the direct effects of renalase on satellite cells, and its physiological role in these cells remains unclear.

The aim of this study was to elucidate the role of renalase in satellite cell proliferation. The results indicate that renalase knockdown inhibits satellite cell proliferation by decreasing Akt–mTOR and ERK1/2 signaling pathways.

## Materials and methods

### Animals

Male C57BL/6 J mice aged 8–12 weeks were used in this study. All mice were housed under a 12 h light/dark cycle at a controlled ambient temperature of 21–26 °C, and they were given standard feed and water.

### Myofiber and satellite cell isolation and culture

The extensor digitorum longus (EDL) muscles were isolated after euthanasia by cervical dislocation and digested in 0.2% type I collagenase at 37 °C for 2 h. The digested muscles were transferred to 5% BSA-coated petri dishes, and single myofibers were isolated under a stereomicroscope using a pasteur pipette. The isolated myofibers were cultured on matrigel-coated dishes. Satellite cells were then cultured in growth medium composed of glucose-free DMEM supplemented with 30% FBS, 1% GlutaMAX, 1% chicken embryo extract, 1% penicillin–streptomycin, and 10 ng/mL bFGF at 37 °C with 5% CO₂ [32].

### Production of adenoviral vectors and cell infection

Adenoviral vectors expressing shRNA targeting renalase (sh-Rnls; VB900138-9658acm) or a scrambled shRNA control (sh-NC; VB010000-0021kwc) were purchased from VectorBuilder (Chicago, IL, USA). These vectors were transfected into 293A cells (R70507; lot# 2,475,537; Thermo Fisher Scientific, Waltham, MA, USA) using PEI MAX (Polysciences, Warrington, PA, USA) to produce recombinant adenoviruses. Viral particles were purified using cesium chloride (CsCl) density gradient ultracentrifugation, and the viral titer was determined using an Adeno-X™ Rapid Titer Kit (Takara Bio, Shiga, Japan) according to the manufacturer’s instructions.

For renalase knockdown, satellite cells were seeded in 12- or 24-well plates at a density of 1.0 × 10^4^ or 2.0 × 10^4^ cells per well, respectively, and infected with adenoviruses at a multiplicity of infection (MOI) of 50. After 12 h, the medium was replaced with fresh growth medium (defined as day 0), and the cells were cultured for up to 4 days.

### Analysis of cell proliferation

Cells were stained with DAPI or Ki67 as a proliferation marker to analyze cell proliferation. EdU incorporation was detected using the Click-iT™ EdU Cell Proliferation Kit for Imaging with Alexa Fluor™ 488 dye (Thermo Fisher Scientific, Waltham, MA, USA). On day 3 of culture, cells were incubated with 10 µM EdU for 6 h and fixed with 4% paraformaldehyde (PFA) for 15 min.

### Immunostaining

Cells were fixed with 2% or 4% PFA for 10 min and blocked/permeabilized for 30 min at room temperature in PBS containing 0.3% Triton X-100 and either 5% goat serum or 2% BSA. The cells were incubated with primary antibodies overnight at 4 °C, and subsequently incubated with fluorescence-conjugated secondary antibodies for 1 h at room temperature. Nuclei were counterstained with DAPI. Fluorescence images were captured using a BZ-X810 microscope (Keyence, Osaka, Japan), and image analysis was performed using the Hybrid Cell Count application (BZ-H4C; Keyence, Osaka, Japan). The antibodies are listed in Supplementary Table 1.

### Western blotting

Cells were lysed in NP-40 lysis buffer (50 mM Tris–HCl, pH 7.4; 150 mM NaCl; 1% NP-40; 1 mM EDTA) supplemented with a protease inhibitor cocktail (FUJIFILM Wako Pure Chemical Corporation, Osaka, Japan) and PhosSTOP (Roche, Basel, Switzerland). The lysates were centrifuged at 12,000 × *g* for 15 min at 4 °C, and the supernatants were collected. The TaKaRa BCA Protein Assay Kit (Takara Bio, Shiga, Japan) was used to determine protein concentrations according to the manufacturer’s instructions. Cell lysates were mixed with 4 × Laemmli sample buffer (62.5 mM Tris–HCl, pH 6.8; 1% SDS; 10% glycerol; 0.005% bromophenol blue; 10% 2-mercaptoethanol) at a ratio of 1:3 and incubated at 95 °C for 5 min.

Equal amounts of protein were separated by Sodium Dodecyl Sulfate–Polyacrylamide Gel Electrophoresis (SDS–PAGE) and transferred to polyvinylidene difluoride membranes. The membranes were blocked with Blocking One (Nacalai Tesque, Kyoto, Japan) or with Tris-buffered saline containing 0.1% Tween 20, 5% skim milk, and 5% Blocking One for 1 h at room temperature. After blocking, the membranes were subsequently incubated with primary antibodies overnight at 4 °C, followed by incubation with HRP-conjugated secondary antibodies for 1 h at room temperature. The blots were developed using EzWestLumi Plus (ATTO, Tokyo, Japan) and analyzed with a FUSION imaging system (Vilber Lourmat, Marne-la-Vallée, France). Antibodies are listed in Supplementary Table 1.

### Quantitative reverse transcription PCR (qRT–PCR)

Total RNA was extracted from cultured cells using the NucleoSpin RNA (Takara Bio, Shiga, Japan). The RNA purity and concentration were measured using a NanoDrop OneC spectrophotometer (Thermo Fisher Scientific, Waltham, MA, USA). Using the ReverTra Ace qPCR RT Master Mix (TOYOBO, Osaka, Japan), cDNA was synthesized from 500 ng of total RNA according to the manufacturer’s instructions. Quantitative PCR was performed using TB Green Premix Ex Taq II (Takara Bio, Shiga, Japan) on a QuantStudio 5 real-time PCR system (Applied Biosystems, Waltham, MA, USA). The mRNA expression level was normalized to that of the housekeeping gene *hypoxanthine phosphoribosyltransferase 1* (*Hprt1*), and relative mRNA expression was calculated using the 2^–ΔΔCt^ method [33]. The primer sequences are listed in Supplementary Table 2.

### Statistics

All data are presented as the mean ± standard error of the mean (SEM). Statistical analyses were performed using GraphPad Prism version 10.6 (GraphPad Software, San Diego, CA, USA). The differences between the two groups were analyzed using a Student's *t* test. Two-way analysis of variance (two-way ANOVA) was used to examine the effects of two factors (time and knockdown). When a significant interaction was detected, Tukey’s multiple comparison test was performed. *P* < 0.05 was considered statistically significant.

## Results

### Renalase knockdown inhibits satellite cell proliferation

**Fig. 1:**
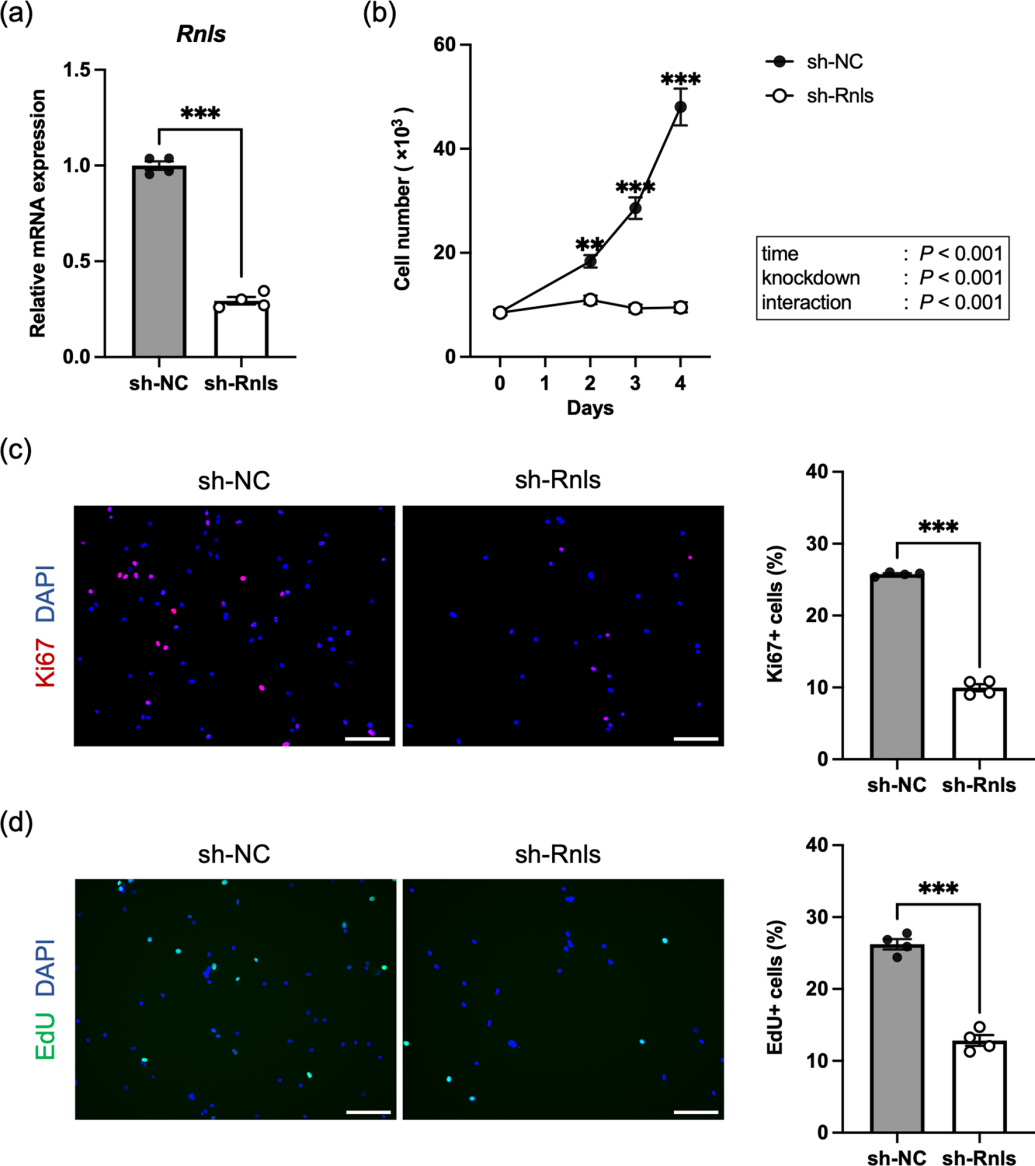
Effects of renalase knockdown on satellite cell proliferation. (**a**) Relative renalase mRNA expression in satellite cells three days after adenoviral infection. (**b**) Cell growth curves from day zero to day four following adenoviral infection. (**c**) Representative immunofluorescence images and quantitative analysis of Ki67-positive cells at day three. (**d**) Representative immunofluorescence images and quantitative analysis of EdU-positive cells at day three. Data are shown as mean ± SEM (n = 4). Scale bars, 100 μm. Statistical significance was determined using Student’s *t*-test for panels (**a**), (**c**), and (**d**), and Tukey’s multiple comparison test for panel (**b**). ***P < 0.001 vs. sh-NC

Satellite cells were infected with adenoviral vectors expressing shRNA targeting renalase to knockdown renalase expression. Renalase mRNA expression was reduced by approximately 70% in sh-Rnls compared with sh-NC three days after adenoviral infection (Fig. 1a). To evaluate the renalase knockdown effects on satellite cell proliferation, cells were cultured for four days after adenoviral infection, and the total cell numbers were quantified based on DAPI staining. Cell number increased progressively in sh-NC, whereas cell proliferation was significantly suppressed in sh-Rnls (Fig. 1b). Consistent with these findings, the proportion of Ki67-positive cells was significantly lower in sh-Rnls compared with sh-NC (Fig. 1c). In addition, the percentage of EdU-positive cells was also significantly reduced in sh-Rnls compared with sh-NC (Fig. 1d).

### Renalase knockdown reduces activated and proliferating satellite cell populations

**Fig. 2:**
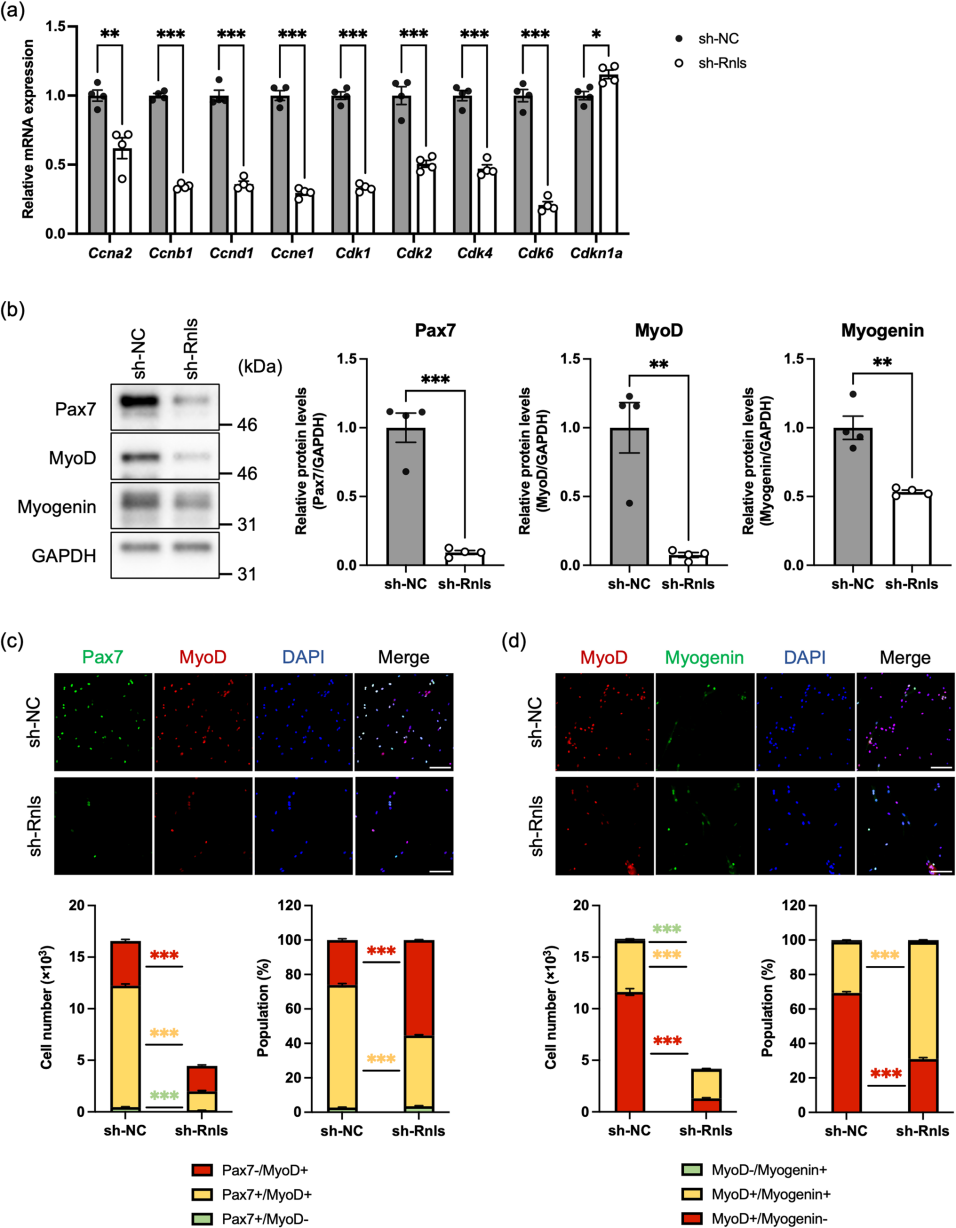
Effects of renalase knockdown on satellite cell populations. (**a**) Relative mRNA expression levels of cell cycle–related genes in satellite cells at day three. (**b**) Representative immunoblots and quantitative analysis of Pax7, MyoD, and Myogenin protein expression in satellite cells at day three. (**c**) Representative immunofluorescence images and quantitative analysis of satellite cells stained for Pax7 and MyoD at day three. (**d**) Representative immunofluorescence images and quantitative analysis of satellite cells stained for MyoD and Myogenin at day three. Data are shown as mean ± SEM (n = 4). Scale bars, 100 μm. Statistical significance was determined using Student’s *t*-test. ***P < 0.001, **P < 0.01, *P < 0.05 vs. sh-NC

Following the observation that renalase knockdown suppressed satellite cell proliferation, the expression of cell cycle–related genes and myogenic markers were examined. The mRNA expression levels of cyclins and cyclin-dependent kinases (CDKs) were significantly reduced and the expression of the cell cycle inhibitor p21 was significantly increased in sh-Rnls compared with sh-NC (Fig. 2a). The protein levels of the quiescence marker Pax7, the activation marker MyoD, and the myogenic differentiation marker Myogenin were significantly decreased in sh-Rnls compared with sh-NC (Fig. 2b).

To further define the myogenic states of satellite cells, double immunofluorescence staining was performed for Pax7/MyoD and MyoD/Myogenin. Pax7⁺/MyoD⁻ cells reflected a quiescent state, Pax7⁺/MyoD⁺ cells reflected an activated/proliferating state, and Pax7⁻/MyoD⁺ cells reflected a myogenic-committed state. In addition, MyoD⁺/Myogenin⁻ cells reflected an activated/proliferating state, MyoD⁺/Myogenin⁺ cells reflected a myogenic-committed state, and MyoD⁻/Myogenin⁺ cells reflected a differentiated state [32]. The number of cells in each myogenic state were significantly reduced in sh-Rnls compared with sh-NC (Fig. 2c, d). Notably, the populations of activated and proliferating satellite cells, including Pax7⁺/MyoD⁺ and MyoD⁺/Myogenin⁻ cells, were markedly decreased in sh-Rnls.

### Renalase knockdown suppresses ERK1/2 and Akt phosphorylation in satellite cells

**Fig. 3:**
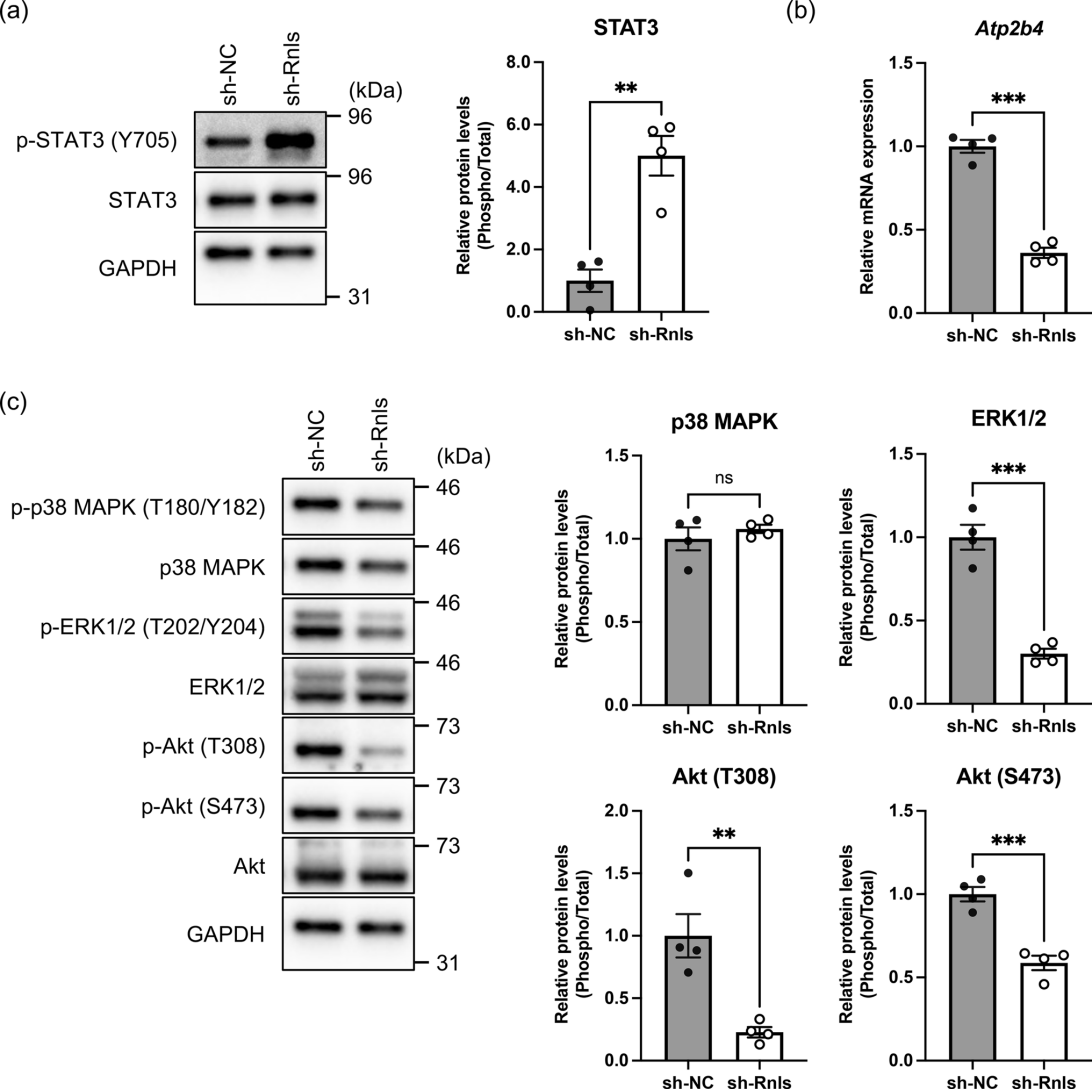
Effects of renalase knockdown on intracellular signaling pathways in satellite cells. (**a**) Representative immunoblots and quantitative analysis of STAT3 phosphorylation in satellite cells at day three. (**b**) Relative mRNA expression level of PMCA4b (*Atp2b4*) in satellite cells at day three. (**c**) Representative immunoblots and quantitative analysis of p38 MAPK, ERK1/2, and Akt phosphorylation in satellite cells at day three. Data are shown as mean ± SEM (n = 4). Statistical significance was determined using Student’s *t*-test. ***P < 0.001, **P < 0.01 vs. sh-NC; ns, not significant

Renalase has been reported to promote cell proliferation through STAT3 signaling in cancer cells; Therefore, we assessed STAT3 phosphorylation. Phosphorylation of STAT3 was significantly increased in sh-Rnls compared with sh-NC (Fig. 3a). In addition, renalase has been reported to activate p38 MAPK, ERK1/2, and Akt signaling through plasma membrane Ca^2^⁺-ATPase 4b (PMCA4b). The mRNA expression level of PMCA4b was significantly reduced in sh-Rnls compared with sh-NC (Fig. 3b). Furthermore, the phosphorylation levels of ERK1/2 and Akt at Thr308 and Ser473 were significantly reduced in sh-Rnls compared with sh-NC, whereas no significant difference was observed in the phosphorylation level of p38 MAPK between sh-Rnls and sh-NC (Fig. 3c).

### Renalase knockdown suppresses mTOR signaling in satellite cells

**Fig. 4:**
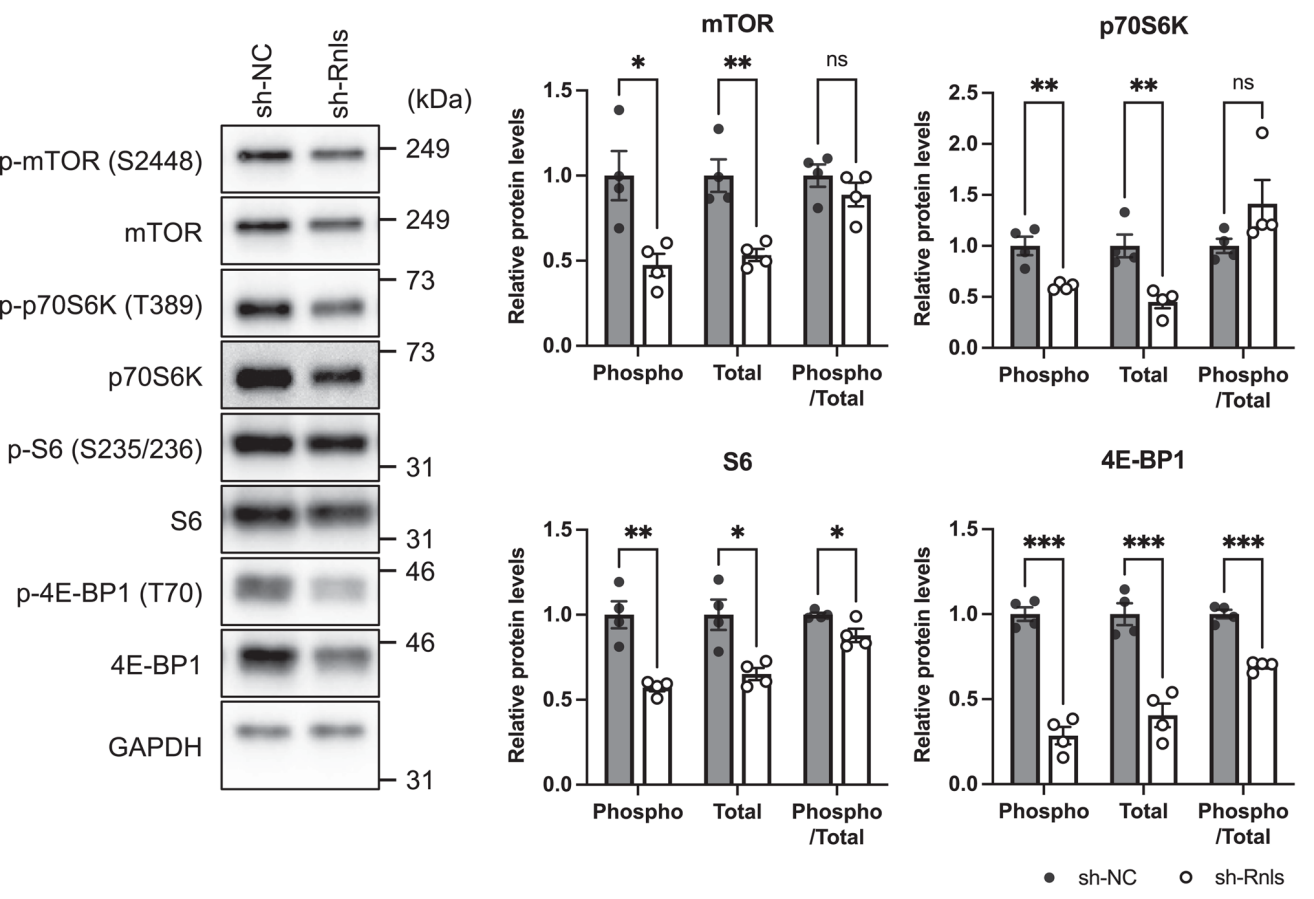
Effects of renalase knockdown on mTOR signaling pathways in satellite cells. Representative immunoblots and quantitative analysis of mTOR, p70S6K, S6, and 4E-BP1 phosphorylation and the total protein expression in satellite cells at day three. Data are shown as mean ± SEM (n = 4). Statistical significance was determined using Student’s *t*-test. ***P < 0.001, **P < 0.01, *P < 0.05 vs. sh-NC; ns, not significant

Following the observation that renalase knockdown attenuated Akt phosphorylation, mTOR signaling pathways downstream of Akt were examined. The phosphorylation levels of mTOR, p70 S6 kinase (p70S6K), ribosomal protein S6, and eukaryotic translation initiation factor 4E–binding protein 1 (4E-BP1) were significantly reduced in sh-Rnls compared with sh-NC (Fig. 4). In addition, the total protein levels of mTOR, p70S6K, S6, and 4E-BP1 were also significantly decreased in sh-Rnls compared with sh-NC (Fig. 4).

## Discussion

Previous studies have reported that renalase expression in skeletal muscle is increased by exercise [28, 29]. However, the physiological role of exercise-induced renalase, particularly its role in satellite cells, has remained unclear. In this study, we investigated the role of renalase in satellite cell proliferation using a renalase knockdown approach. The results indicate that renalase knockdown significantly inhibits satellite cell proliferation by decreasing Akt–mTOR and ERK1/2 signaling pathways.

Renalase knockdown markedly inhibited satellite cell proliferation. It was accompanied by decreased Akt–mTOR and ERK1/2 signaling. Akt is known to regulate cell growth by promoting protein synthesis through mTOR-mediated phosphorylation of downstream targets such as p70S6K and 4EBP1 [34, 35]. Previous studies have shown that Akt1 deletion or inhibition of mTOR signaling in satellite cells suppresses their proliferation [36, 37]. In addition, ERK1/2 plays a critical role of cell-cycle progression from the G1 to S phase [9], and its inactivation or pharmacological inhibition induces cell-cycle exit and reduces cell proliferation [9, 38]. Therefore, these findings suggest that the suppression of satellite cell proliferation observed following renalase knockdown is primarily attributable to the downregulation of Akt–mTOR and ERK1/2 signaling.

In tumor cells, the inhibition of renalase has been reported to suppress cell proliferation by reducing the phosphorylation of STAT3 [25, 26]. In contrast, in this study, renalase knockdown suppressed satellite cell proliferation while the phosphorylation of STAT3 was increased, indicating a cellular response distinct from that observed in tumor cells. This discrepancy suggests that the regulatory mechanisms by which renalase modulates STAT3 signaling differ between tumor and non-tumor cells, or among cell types. Although renalase has been reported to not affect the phosphorylation of STAT3 in macrophages [25], this supports the notion that renalase-mediated STAT3 regulation is context dependent. Furthermore, the role of STAT3 in satellite cell proliferation remains controversial, with reports demonstrating both inhibitory [39–42] and promotive [43, 44] effects. Therefore, further investigation is required to clarify the relationship between enhanced the phosphorylation of STAT3 and reduced satellite cell proliferation observed in this study.

To determine whether the suppression of proliferation induced by renalase knockdown was attributable to premature differentiation or to an intrinsic impairment of cell proliferation, the myogenic states were assessed based on Pax7, MyoD, and Myogenin expression by immunostaining [32]. Renalase knockdown reduced the number of cells across all myogenic states, with a particularly pronounced decrease in Pax7⁺/MyoD⁺ and MyoD⁺/Myogenin⁻ populations, which represent activated and proliferating satellite cells. In addition, the numbers of Ki67-positive and EdU-positive cells were significantly reduced, and the expression of genes involved in cell-cycle progression, including cyclins and cyclin-dependent kinases, were significantly decreased. Therefore, these findings indicate that renalase knockdown suppresses satellite cell proliferation not by promoting differentiation, but by directly impairing cell proliferation.

Satellite cells possess self-renewal capacity and maintain their pool by returning to a quiescent Pax7⁺/MyoD⁻ state following proliferation [4, 5]. In this study, renalase knockdown also reduced the Pax7⁺/MyoD⁻ cell population. This finding suggests that renalase contributes to the maintenance of the satellite cell pool by ensuring proper progression of the activation–proliferation cycle. Considering the central role of satellite cells in skeletal muscle regeneration [1–3], reduced renalase expression may impair both cell proliferation and self-renewal, ultimately leading to defective muscle regeneration.

A limitation of our study is that we were unable to confirm renalase protein expression in satellite cells, as none of the commercially available anti-renalase antibodies we tested produced specific signals in mouse samples by Western blotting. Future studies could address this limitation to further elucidate the role of renalase and its interactions with other proteins.

## Conclusion

This study reveals that renalase is a critical factor for maintaining satellite cell proliferation by activating the Akt–mTOR and ERK1/2 signaling pathways. Furthermore, these findings suggest that renalase contributes to the maintenance of the satellite cell pool by ensuring proper progression of the satellite cell activation–proliferation cycle.

## Supplementary Information

Below is the link to the electronic supplementary material.

Supplementary file1 (PDF 16 KB) Supplementary Table 1. List of antibodies

Supplementary file2 (PDF 13 KB) Supplementary Table 2. Primer sequences used for qRT-PCR

## Data Availability

Raw data are available from the corresponding author upon reasonable request.
