# Correction: Renalase knockdown inhibits proliferation of mouse satellite cells

**DOI:** 10.1007/s11033-026-12046-9

**Published:** 2026-06-10

**Authors:** Yuri Kato, Katsuyuki Tokinoya, Kai Aoki, Kazuhiro Takekoshi

**Affiliations:** 1 Graduate School of Comprehensive Human Sciences, University of Tsukuba, 1-1-1 Tennodai, Tsukuba, Ibaraki 305-8574 Japan; 2 Research Fellowship for Young Scientists, Japan Society for the Promotion of Science, 5-3-1 Kojimachi, Chiyodaku, Tokyo 102-0083 Japan; 3 College of Education, Yokohama National University, 79-2 Tokiwadai, Hodogaya-Ku, Yokohama, Kanagawa 240-8501 Japan; 4 Department of Health and Nutrition, Faculty of Health Science, Niigata University of Health and Welfare, 1398 Shimami-Cho, Kita-Ku, Niigata, 950-3198 Japan; 5 Faculty of Medicine, Institute of Medicine, University of Tsukuba, 1-1-1 Tennodai, Tsukuba, Ibaraki 305-8574 Japan

**Correction to: Molecular Biology Reports (2026) 53:637**

10.1007/s11033-026-11803-0

In this article, Fig. 3 appeared incorrectly and has now been corrected in the original publication. For completeness and transparency, the correct and old incorrect versions are displayed below.

Incorrect Figure 3

**Fig. 3:**
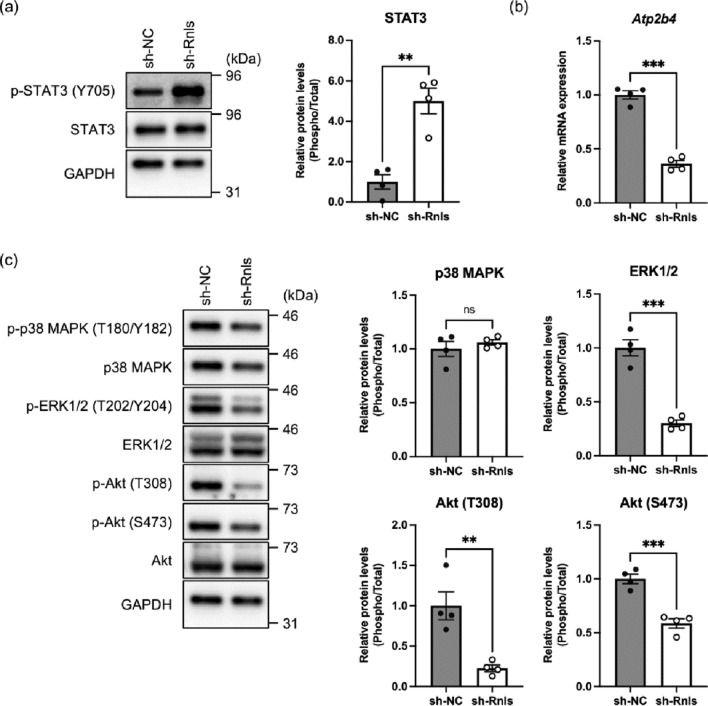
Effects of renalase knockdown on intracellular signaling pathways in satellite cells. (**a**) Representative immunoblots and quantitative analysis of STAT3 phosphorylation in satellite cells at day three. (**b**) Relative mRNA expression level of PMCA4b (Atp2b4) in satellite cells at day three. (**c**) Representative immunoblots and quantitative analysis of p38 MAPK, ERK1/2, and Akt phosphorylation in satellite cells at day three. Data are shown as mean ± SEM (n = 4). Statistical significance was determined using Student’s t-test. ***P < 0.001, **P < 0.01 vs. sh-NC; ns, not significant

Correct Figure 3

**Fig. 3:**
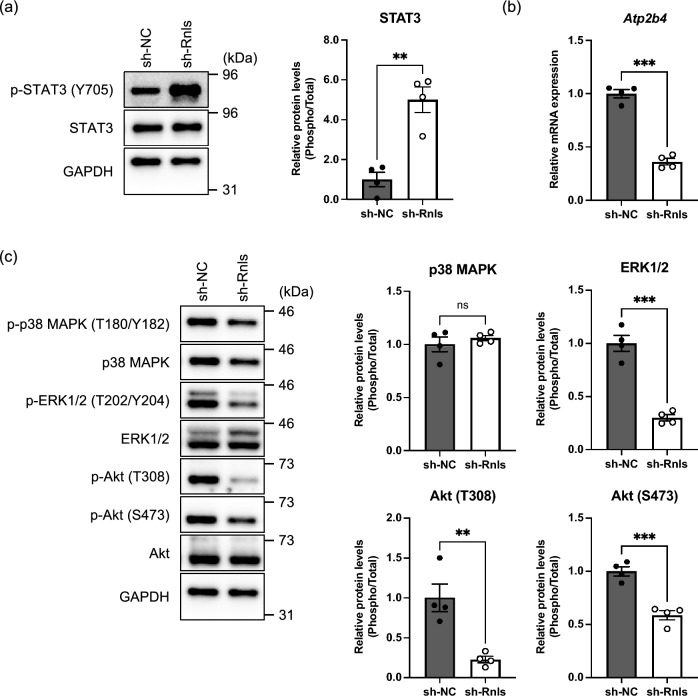
Effects of renalase knockdown on intracellular signaling pathways in satellite cells. (**a**) Representative immunoblots and quantitative analysis of STAT3 phosphorylation in satellite cells at day three. (**b**) Relative mRNA expression level of PMCA4b (Atp2b4) in satellite cells at day three. (**c**) Representative immunoblots and quantitative analysis of p38 MAPK, ERK1/2, and Akt phosphorylation in satellite cells at day three. Data are shown as mean ± SEM (n = 4). Statistical significance was determined using Student’s t-test. ***P < 0.001, **P < 0.01 vs. sh-NC; ns, not significant

Additionally, Supplementary file 3 has been removed.

The original article has been corrected.

