# Supplementary material for: Renalase knockdown inhibits proliferation of mouse satellite cells
Source: Mol Biol Rep. 2026 Apr 17;53(1):637. doi: 10.1007/s11033-026-11803-0 (PMC13090268; doi:10.1007/s11033-026-11803-0)
Supplement: Supplementary file 1 — Supplementary file1 (PDF 16 KB) Supplementary Table 1. List of antibodies [file 11033_2026_11803_MOESM1_ESM.pdf]

Supplementary Table 1. List of antibodies

Antibodies	Source	Catalog number
Anti-Ki67	Abcam	ab16667
Anti-Pax7	Santa Cruz	sc-81648
Anti-MyoD	Santa Cruz	sc-377460
Anti-Myogenin	Santa Cruz	sc-12732
Anti-Phospho-p38 MAPK (Thr180/Tyr182)	Cell Signaling Technology	#4511
Anti-p38 MAPK	Cell Signaling Technology	#9212
Anti-Phospho-p44/42 MAPK (Erk1/2) (Thr202/Tyr204)	Cell Signaling Technology	#4370
Anti-p44/42 MAPK (Erk1/2)	Cell Signaling Technology	#4695
Anti-Phospho-Akt (Thr308)	Cell Signaling Technology	#5106
Anti-Phospho-Akt (Ser473)	Cell Signaling Technology	#9271
Anti-Akt	Cell Signaling Technology	#9272
Anti-Phospho-Stat3 (Tyr705)	Cell Signaling Technology	#9145
Anti-Stat3	Cell Signaling Technology	#4904
Anti-Phospho-mTOR (Ser2448)	Cell Signaling Technology	#5536
Anti-mTOR	Cell Signaling Technology	#2983
Anti-Phospho-p70 S6 Kinase (Thr389)	Cell Signaling Technology	#9234
Anti-p70 S6 Kinase	Cell Signaling Technology	#9202
Anti-Phospho-S6 Ribosomal Protein (Ser235/236)	Cell Signaling Technology	#2211
Anti-S6 Ribosomal Protein	Cell Signaling Technology	#2217
Anti-Phospho-4E-BP1 (Thr70)	Cell Signaling Technology	#9455
Anti-4E-BP1	Cell Signaling Technology	#9452
Anti-GAPDH	Santa Cruz	sc-365062
Alexa Fluor 594 AffiniPure Goat Anti-Rabbit IgG (H+L)	Jackson ImmunoResearch	111-585-003
Goat anti-Mouse IgG1 Cross-Adsorbed Secondary Antibody, Alexa Fluor 488	Thermo Fisher Scientific	A-21121
Goat anti-Mouse IgG2b Cross-Adsorbed Secondary Antibody, Alexa Fluor 594	Thermo Fisher Scientific	A-21145
Anti-rabbit IgG, HRP-linked Antibody	Cell Signaling Technology	#7074
Anti-mouse IgG, HRP-linked Antibody	Cell Signaling Technology	#7076
