# Supplementary material for: Renalase knockdown inhibits proliferation of mouse satellite cells
Source: Mol Biol Rep. 2026 Apr 17;53(1):637. doi: 10.1007/s11033-026-11803-0 (PMC13090268; doi:10.1007/s11033-026-11803-0)
Supplement: Supplementary file 2 — Supplementary file2 (PDF 13 KB) Supplementary Table 2. Primer sequences used for qRT-PCR [file 11033_2026_11803_MOESM2_ESM.pdf]

Supplementary Table 2. Primer sequences used for qRT-PCR

Gene	Forward (5' – 3')	Reverse (5' – 3')
Hprt1	GGACTGATTATGGACAGGACTGA	TGTAATCCAGCAGGTCAGCA
Rnls	TGAAGCCTCTGACATCCCCC	AGGGAGACTTCTGCACCTGAC
Ccna2	TCTGGGATTAAAGGCGCCAC	CTGCTGCTCAGTGGATCTGT
Ccnb1	GCCTCACAAAGCACATGACTG	GTACAGTTCAGCTGTGCCA
Ccnd1	CAGCCCCAACAACTTCCTCT	CAGGGCCTTGACCGGG
Ccne1	GGCAAATGTGGCCGTGTTTT	GCTGACTGCTATCCTCGCTT
Cdk1	GTGTACACACACGAGGTAGTGA	ATCTCTGAGTCGCCGTGGA
Cdk2	AAGGTGGAGAAGATTGGAGAGG	ACACCTTCAGTCTCAGTGTCG
Cdk4	CCTCAAGAGTGTGAGAGTTCCT	GACATCCATCAGCCGTACAAC
Cdk6	CTTACCTCGGTGGTCGTCAC	CCACGTCTGAACTTCCACGA
Cdkn1a	TATCCAGACATTCAGAGCCACAG	ATGAGCGCATCGCAATCAC
Atp2b4	GACGAGATTGACCTTGCCGA	AGGTGTGGATGGAGCTACGA
